# Study on the influence of microabscess on the recurrence of granulomatous lobular mastitis and stratified treatment strategies

**DOI:** 10.1371/journal.pone.0358780

**Published:** 2026-09-23

**Authors:** Jin Chen, Jiefang Dai, Ruiyang Wu, Haiyan Zhang

**Affiliations:** 1 Breast and Thyroid Department, Sichuan Provincial Maternity and Child Health Care Hospital, Sichuan Provincial Women’s and Children’s Hospita, The Affiliated Women’s and Children’s Hospital of Chengdu Medical College, Chengdu, China; 2 Ultrasound Department, Sichuan Provincial Maternity and Child Health Care Hospital, Sichuan Provincial Women’s and Children’s Hospital, The Affiliated Women’s and Children’s Hospital of Chengdu Medical College, Chengdu, China; 3 Breast Center, West China Second University Hospital, Sichuan University, Chengdu, China; Children’s National Hospital, George Washington University, UNITED STATES OF AMERICA

## Abstract

**Objective:**

We aimed to clarify whether microabscess observed on pathological biopsy independently predicts recurrence in granulomatous lobular mastitis (GLM), and to compare recurrence risks across different treatment regimens stratified by microabscess status. Our findings may help clinicians design individualized anti-recurrence therapy for GLM patients.

**Methods:**

This single-center retrospective cohort enrolled 520 women with pathologically confirmed GLM from a non-puerperal mastitis registry maintained at a provincial maternal and child health hospital. Patient records spanned January 2017 to December 2024, with all follow-up completed September 1, 2025. We collected demographic, laboratory, ultrasound, medication and surgical data for all participants. Cox proportional hazards regression models identified independent recurrence predictors. Kaplan–Meier curves with log-rank tests were used to compare 1-year recurrence rates between patients with and without histologic microabscess. Subgroup analyses further tested whether quinolone use or surgical resection modified the prognostic impact of microabscess.

**Results:**

The cohort’s average age at GLM onset was 31.5 years; 268 patients (51.5%) had microabscess on histology, while 252 (48.5%) did not. Multivariate Cox regression identified affected breast laterality, histologic microabscess (HR = 0.605, 95% CI 0.413–0.886, P = 0.010), peripheral white blood cell count, anti-mycobacterial therapy, and corticosteroid use as independent recurrence predictors. Microabscess acted as a protective factor against relapse. The 1-year recurrence rate was lower in patients with microabscess (20.1%) than those without (28.6%, P = 0.021). Baseline comparisons revealed higher rates of quinolone administration and complete surgical excision among microabscess-positive individuals (P = 0.007 and P = 0.004, respectively). When restricted to patients receiving quinolones, the recurrence gap between microabscess-positive and negative cases widened significantly (19.7% vs 28.7%, P = 0.030); no meaningful intergroup difference emerged for patients who did not take quinolones (P = 0.432). Anti-mycobacterial medication reduced recurrence risk regardless of microabscess status, while corticosteroid use raised relapse risk. Abscess drainage only lowered 1-year recurrence for patients with microabscess (17.4% vs 27.0%, P = 0.036), with no measurable benefit for those without microabscess. Surgical resection, cephalosporins and bromocriptine showed no independent ability to reduce recurrence in either subgroup.

**Conclusions:**

Histologically confirmed microabscess independently lowers recurrence risk in GLM. For patients with microabscess, a combined regimen of quinolones, abscess drainage and anti-mycobacterial therapy yields the best anti-relapse outcomes. Corticosteroid use markedly increases recurrence likelihood. Treatment decisions for GLM should be adjusted based on whether microabscess is present on pathology.

## Introduction

Granulomatous lobular mastitis (GLM) is a chronic inflammatory breast disorder almost exclusively seen in women of childbearing age who have previously given birth. The disease forms non-caseating granulomas centered on breast lobules, and patients typically present with painful hard masses, recurrent abscesses, or persistent cutaneous fistulas. GLM progresses slowly and relapses frequently, and its imaging appearance often overlaps with breast cancer, leading to frequent misdiagnosis in routine breast clinics [1]. Domestic case numbers have climbed steadily over the past decade, yet its exact pathogenesis remains unclear. Existing theories link GLM onset to autoimmune dysfunction, elevated prolactin, hyperlipidemia, ductal injury, and infection with Corynebacterium species, often acting together [2,3]. It should be noted that Corynebacterium species are common commensal components of human cutaneous microbiota. Therefore, isolating Corynebacterium from breast tissue or secretion samples cannot automatically confirm a causal pathogenic role for GLM. Conventional bacterial culture approaches are time‑consuming and have limited diagnostic sensitivity. Rapid molecular detection strategies, such as metabolism‑driven sensor arrays, may help improve pathogen identification and clinical interpretation of Corynebacterium‑associated mastitis [4,5].

No universal treatment protocol exists for GLM globally. Clinicians rely on variable combinations of broad-spectrum antibiotics, corticosteroids, anti-mycobacterial drugs, bromocriptine, percutaneous drainage, and full surgical resection. Published recurrence rates range from 15% to 40% across different treatment centers [6]. Relapse places substantial physical and psychological burden on patients, prolongs care cycles, and increases risk of permanent breast deformity. Identifying reliable pathological markers to guide targeted therapy has therefore become a key research priority [7].

Microabscesses are defined as microscopic collections of neutrophils forming tiny pus cavities within lobules or stroma on histologic slides; these lesions cannot be palpated on clinical exam and are distinct from large macroscopic breast abscesses. They represent a secondary inflammatory change during active GLM [8]. Most existing GLM risk models focus on clinical variables such as lesion size, bilateral disease, high prolactin, and steroid exposure. Few large cohorts have assessed how histologic microabscess correlates with recurrence, and prior work has not developed stratified treatment plans tailored to microabscess status [9].

Two domestic consensus documents touch on abscess management but lack granular guidance for microscopic lesions. The 2016 Chinese expert consensus on non-puerperal mastitis notes that abscess formation alters treatment planning, without separating microabscess from macroscopic abscess cases [10]. The 2021 Hunan GLM consensus confirms drainage relieves acute inflammation but does not examine long-term recurrence differences between patients with and without microabscess [11]. Small foreign cohorts have suggested microscopic pus collections may signal localized inflammation and lower pathogen burden, but no Chinese dataset exceeding 500 patients has validated this idea, nor have studies tested how mainstream drugs (quinolones, anti-mycobacterials, steroids) interact with microabscess to shape relapse risk [12].

We built our analysis around a standardized non-puerperal mastitis database housed at a provincial women’s hospital, enrolling 520 pathologically verified GLM patients with complete follow-up records. Cox regression models quantified microabscess’s independent prognostic weight, and Kaplan–Meier curves compared 1-year relapse rates between microabscess subgroups. We then ran stratified survival analyses to assess how antibiotics, drainage, surgical resection, anti-mycobacterials, and corticosteroids perform in each patient subgroup. Our goal was to clarify the prognostic value of histologic microabscess and propose stratified treatment pathways supported by large-cohort real-world data [13].

## Methods

### Study population and data source

In this retrospective observational cohort study, all clinical data were extracted from the electronic medical record system of Sichuan Provincial Maternity and Child Health Care Hospital on January 1, 2026. Data originated from the hospital’s dedicated Non-Puerperal Mastitis Database (NPMD), a registry established in March 2022 within the Department of Breast and Thyroid Surgery, which covered patients diagnosed with non-puerperal mastitis (NPM) receiving inpatient or outpatient treatment in our institution.

The database was dynamically updated: retrospective data of patients diagnosed between January 2017 and February 2022 were collated, while newly diagnosed eligible cases were prospectively enrolled starting March 2022. The complete dataset was retrieved on January 1, 2026, including 710 NPM patients diagnosed from January 2017 to December 2024, with all participants’ follow-up terminated on September 1, 2025. After excluding 44 patients with plasma cell mastitis and another 146 patients with missing histologic microabscess records, a total of 520 pathologically confirmed GLM patients were finally included for analysis.

All clinical treatments adopted for patients strictly complied with domestic Chinese expert consensus on non-puerperal mastitis and international multidisciplinary guidelines for granulomatous lobular mastitis, without extra experimental interventions imposed on patients.

This study was reviewed and approved by the Ethics Committee of Sichuan Provincial Maternity and Child Health Care Hospital (Ethics No. 20220330−024). Given the retrospective design, exclusive use of de-identified routine clinical data and minimal privacy risk, the Ethics Committee granted a full waiver of written informed consent for all enrolled subjects. All patient personal information was fully desensitized before statistical analysis to protect individual privacy, and the research was conducted in accordance with the Declaration of Helsinki and its subsequent amendments.

### Statistical analysis

Continuous variables were expressed as mean ± standard deviation and compared using the Student’s t-test. Categorical variables were summarized as frequencies and percentages, and group comparisons were performed using the Chi-square test.

To determine whether the presence of microabscesses influenced recurrence in patients with GLM, two analytical methods were employed. First, a Cox proportional hazards regression model was performed to identify potential predictors of disease recurrence, with a particular focus on microabscesses. Second, a survival analysis was conducted to compare the one-year recurrence rates between patients with microabscess-positive and microabscess-negative status, in order to evaluate potential differences.

To evaluate the potential influence of microabscesses on recurrence in patients with granulomatous lobular mastitis (GLM), the analysis was conducted in several steps. First, baseline characteristics were compared between patients with and without microabscesses to identify any significant differences. Subsequently, variables showing imbalance between the groups were incorporated into subgroup survival analyses to assess whether they modified the association between microabscess status and recurrence. Finally, to explore optimal treatment strategies for each group, survival analysis was used to compare recurrence rates under different therapeutic regimens between microabscess-positive and microabscess-negative patients.

Kaplan-Meier survival curves were compared between groups using the log-rank test. All hypothesis tests were two-sided, and a p-value of less than 0.05 was considered statistically significant. Statistical analyses were performed using SPSS version 25.0, and survival curves were plotted with Stata version 19.

## Results

### Descriptive characteristics and prognostic factor analysis

A total of 520 patients with GLM were enrolled in this study, all of whom were female and had a mean age of 31.5 years. Univariable and multivariable Cox proportional hazards regression analyses identified the affected side (P < 0.001), microabscess (HR = 0.605, 95% CI: 0.413–0.886, P = 0.010), white blood cell count (P < 0.001), antitubercular therapy (HR = 0.336, 95% CI: 0.213–0.529, P < 0.001), and corticosteroid therapy (HR = 2.568, 95% CI: 1.509–4.370, P = 0.001) as independent risk factors for disease recurrence in GLM patients (Table 1).

**Table 1:** Univariable and multivariate analysis of prognostic factors for 520 patients with GLM.

Factors	N(%)/Mean ± SD	Univariate	Multivariate	
		P value	HR(95%CI)	P value
**Age at diagnosis (years)**	31.5 ± 4.7	0.365	0.972(0.932-1.013)	0.176
**Affected side**		<0.001		<0.001
left	273(52.5)		Ref	
right	236(45.4)		2.076(1.400-3.079)	<0.001
bilateral	11(2.1)		7.864(3.052-20.266)	<0.001
**Days to first visit (days)**	19.8 ± 44.0	0.954	0.997(0.993-1.001)	0.194
**Maximum lesion diameter on ultrasound**		0.747		0.624
< 4 cm	187(36.0)		Ref	
≥ 4 cm	311(59.8)		1.085(0.719-1.637)	0.699
unknown	22(4.2)		0.530(0.115-2.440)	0.415
**Ultrasound-detected lesion count**		0.540		0.378
solitary	65(12.5)		Ref	
multiple	432(83.1)		0.794(0.435-1.449)	0.452
unknown	23(4.4)		2.079(0.449-9.628)	0.349
**Mammary abscess**		0.946		0.531
no	68(13.1)		Ref	
yes	452(86.9)		1.236(0.637-2.401)	
**Microabscess**		0.024		0.010
no	252(48.5)		Ref	
yes	268(51.5)		0.605(0.413-0.886)	
**White blood cell**		<0.001		<0.001
< 10*10^9/L	281(54.0)		Ref	
≥ 10*10^9/L	233(44.8)		2.004(1.309-3.070)	0.001
unknown	6(1.2)		8.191(2.598-25.820)	<0.001
**C-reactive protein**		0.275		0.056
< 10 mg/L	217(41.7)		Ref	
≥ 10 mg/L	152(29.2)		0.934(0.563-1.549)	0.790
unknown	151(29.0)		1.626(1.022-2.586)	0.040
**Prolactin**		0.279		0.989
≤ 650 uIU/ml	356(68.5)		Ref	
> 650 uIU/ml	130(25.0)		1.002(0.603-1.665)	0.994
unknown	34(6.5)		0.940(0.410-2.156)	0.884
**Hyperlipidemia**		0.654		0.774
no	175(33.7)		Ref	
yes	181(34.8)		1.062(0.690-1.633)	0.786
unknown	164(31.5)		0.879(0.525-1.472)	0.624
**Quinolone therapy**		0.629		0.861
no	82(15.8)		Ref	
yes	438(84.2)		0.953(0.556-1.634)	
**Penicillin therapy**		0.333		0.443
no	497(95.6)		Ref	
yes	23(4.4)		0.624(0.187-2.080)	
**Cephalosporin therapy**		0.749		0.482
no	380(73.1)		Ref	
yes	140(26.9)		1.166(0.760-1.790)	
**Macrolide therapy**		0.519		0.991
no	495(95.2)		Ref	
yes	25(4.8)		1.005(0.439-2.300)	
**Nitroimidazole therapy**		0.355		0.048
no	502(96.5)		Ref	
yes	18(3.5)		2.568(1.509-4.370)	
**Antitubercular therapy**		<0.001		<0.001
no	340(65.4)		Ref	
yes	180(34.6)		0.336(0.213-0.529)	
**Corticosteroid therapy**		<0.001		0.001
no	169(32.5)		Ref	
yes	351(67.5)		2.568(1.509-4.370)	
**Bromocriptine therapy**		0.666		0.920
no	271(52.1)		Ref	
yes	249(47.9)		0.979(0.640-1.496)	
**Abscess drainage**		0.085		0.096
no	152(29.2)		Ref	
yes	368(70.8)		0.637(0.375-1.083)	
**Surgical excision**		0.146		0.019
no	77(14.8)		Ref	
yes	443(85.2)		0.517(0.297-0.899)	

### Survival by microabscess status

**Fig 1:**
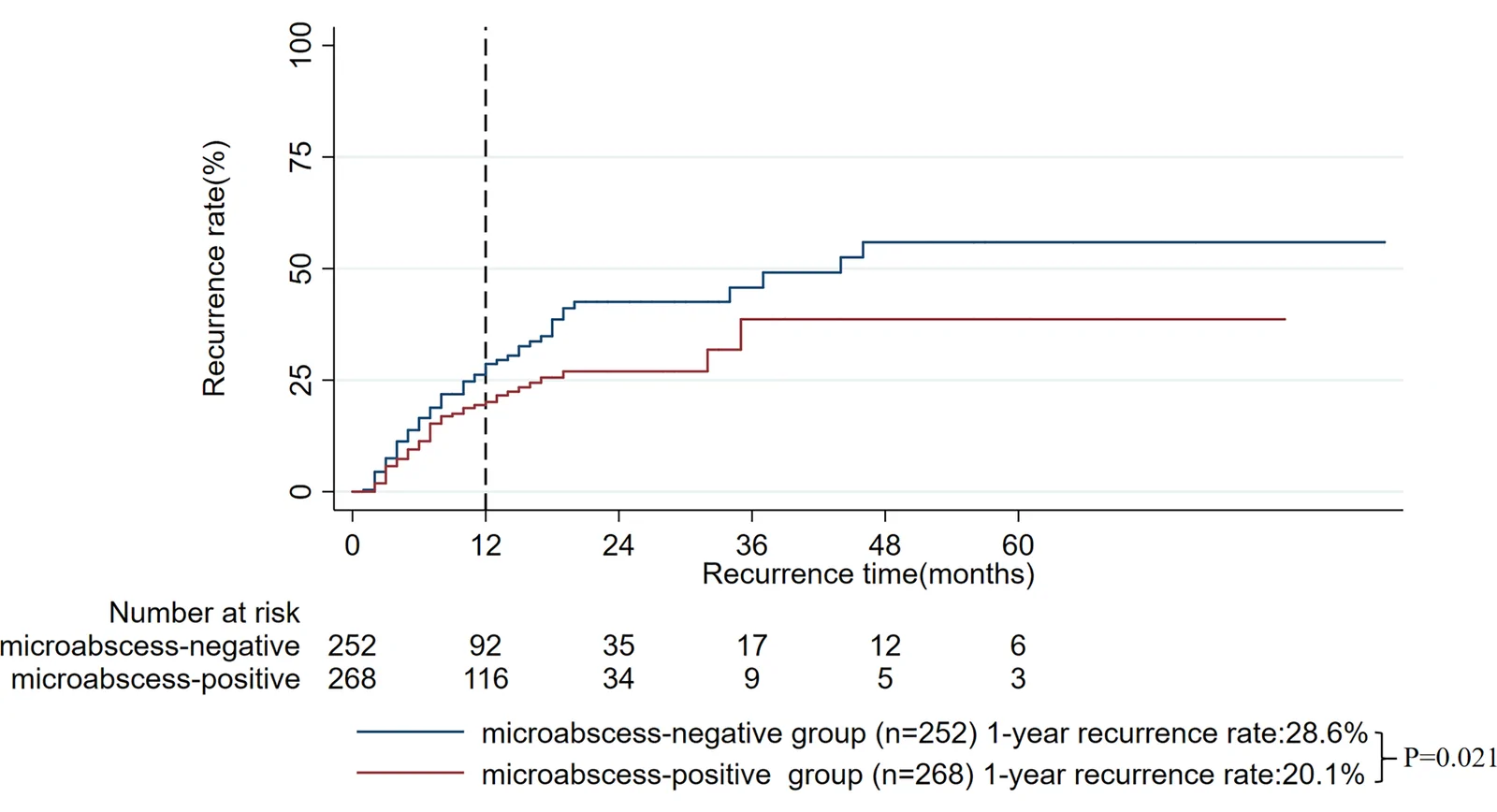
Association between microabscess status and one-year recurrence rate.

To further investigate the impact of microabscesses on recurrence, a survival analysis was conducted. It revealed that the microabscess-positive group had a significantly lower one-year recurrence rate compared to the microabscess-negative group (20.1% vs. 28.6%, P = 0.021, Fig 1), suggesting that the presence of microabscesses may be a potential indicator for lower recurrence risk in GLM patients.

### Baseline characteristics by microabscess status

To further assess whether other factors might confound the impact of microabscesses on prognosis, baseline characteristics were compared between the microabscess-positive and microabscess-negative groups (Table 2). The analysis revealed that the microabscess-positive group had higher rates of quinolone therapy and surgical excision compared to the microabscess-negative group, while no significant differences were observed in other baseline characteristics (all P > 0.05, Table 2).

**Table 2:** Comparison of baseline characteristics between microabscess-positive and microabscess-negative groups.

Factors	microabscess-positive group (N = 268)	microabscess-negative group (N = 252)	P value
	N(%)/x ± s	N(%)/x ± s	
**Age at diagnosis (years)**	31.6 ± 4.3	31.5 ± 5.1	0.742
**Affected side**			0.505
left	147(54.9)	126(50.0)	
right	115(42.9)	121(48.0)	
bilateral	6(2.2)	5(2.0)	
**Days to first visit (days)**	19.9 ± 33.2	19.7 ± 53.2	0.951
**Maximum lesion diameter on ultrasound**			0.354
< 4 cm	100(37.3)	87(34.5)	
≥ 4 cm	154(57.5)	157(62.3)	
unknown	14(5.2)	8(3.2)	
**Ultrasound-detected lesion count**			0.730
solitary	31(11.6)	34(13.5)	
multiple	224(83.6)	208(82.5)	
unknown	13(4.9)	10(4.0)	
**Mammary abscess**			0.594
no	33(12.3)	35(13.9)	
yes	235(87.7)	217(86.1)	
**White blood cell**			0.074
< 10*10^9/L	132(49.3)	149(59.1)	
≥ 10*10^9/L	133(49.6)	100(39.7)	
unknown	3(1.1)	3(1.2)	
**C-reactive protein**			0.605
< 10 mg/L	107(39.9)	110(43.7)	
≥ 10 mg/L	83(31.0)	69(27.4)	
unknown	78(29.1)	73(29.0)	
**Prolactin**			0.531
≤ 650 uIU/ml	182(67.9)	174(69.0)	
> 650 uIU/ml	71(26.5)	59(23.4)	
unknown	15(5.6)	19(7.5)	
**Hyperlipidemia**			0.610
no	92(34.3)	83(32.9)	
yes	88(32.8)	93(36.9)	
unknown	88(32.8)	76(30.2)	
**Quinolone therapy**			0.007
no	31(11.6)	51(20.2)	
yes	237(88.4)	201(79.8)	
**Penicillin therapy**			0.077
no	252(94.0)	245(97.2)	
yes	16(6.0)	7(2.8)	
**Cephalosporin therapy**			0.715
no	194(72.4)	186(73.8)	
yes	74(27.6)	66(26.2)	
**Macrolide therapy**			0.440
no	257(95.9)	238(94.4)	
yes	11(4.1)	14(5.6)	
**Nitroimidazole therapy**			0.729
no	258(96.3)	244(96.8)	
yes	10(3.7)	8(3.2)	
**Antitubercular therapy**			0.551
no	172(64.2)	168(66.7)	
yes	96(35.8)	84(33.3)	
**Corticosteroid therapy**			0.088
no	78(29.1)	91(36.1)	
yes	190(70.9)	161(63.9)	
**Bromocriptine therapy**			0.198
no	147(54.9)	124(49.2)	
yes	121(45.1)	128(50.8)	
**Abscess drainage**			0.796
no	77(28.7)	75(29.8)	
yes	191(71.3)	177(70.2)	
**Surgical excision**			0.004
no	28(10.4)	49(19.4)	
yes	240(89.6)	203(80.6)	

### Subgroup analysis

**Fig 2:**
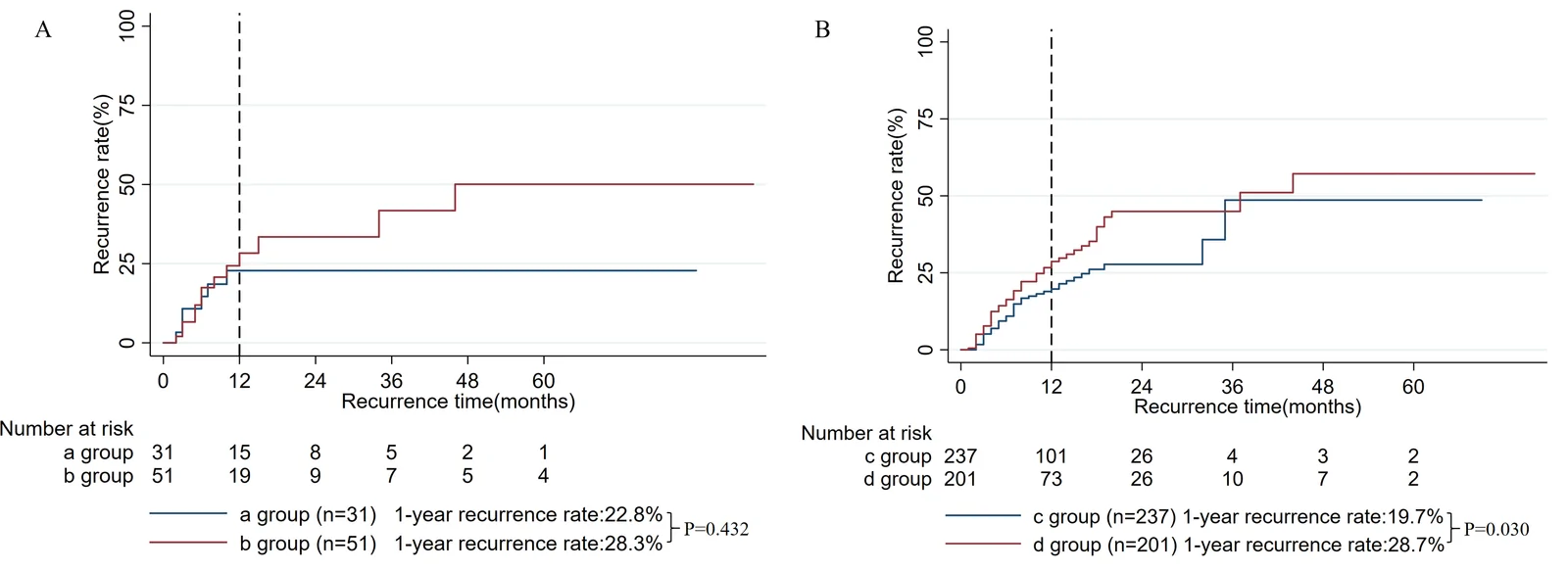
Recurrence‑free survival curves stratified by microabscess status according to quinolone therapy. A. Recurrence-free survival in patients not receiving quinolone therapy, stratified by microabscess status. B: Recurrence-free survival in patients receiving quinolone therapy, stratified by microabscess status.

Since the microabscess-positive group had a higher rate of quinolone therapy compared to the microabscess-negative group (88.4% vs. 79.1%, P = 0.007; Table 2), we further stratified all 520 patients into four subgroups based on microabscess status and quinolone therapy use to investigate whether quinolone therapy influenced the prognostic effect of microabscesses (Table 3). Survival analysis revealed that among patients who did not receive quinolone therapy, the microabscess-positive subgroup (a) had a lower one-year recurrence rate than the microabscess-negative subgroup (b) (22.8% vs. 28.3%), although the difference was not statistically significant (P = 0.432, Fig 2A). In contrast, among patients who received quinolone therapy, the microabscess-positive subgroup (c) showed a significantly lower one-year recurrence rate than the microabscess-negative subgroup (d) (19.7% vs. 28.7%, P = 0.030, Fig 2B).

**Table 3:** Comparison of one-year recurrence rates by microabscess status and quinolone therapy.

group	quinolone therapy	microabscess	N	1-year recurrence rate(%)
a	no	positive	31	22.8
b	no	negative	51	28.3
c	yes	positive	237	19.7
d	yes	negative	201	28.7

**Fig 3:**
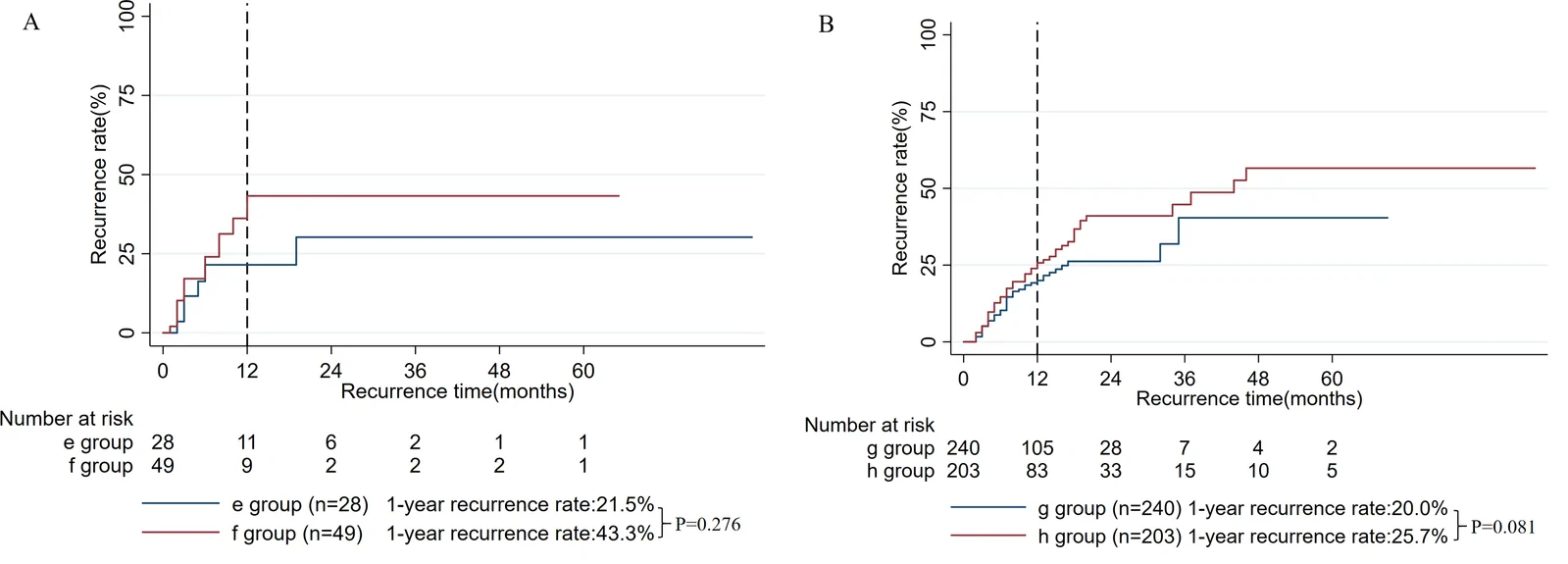
Recurrence‑free survival curves stratified by microabscess status according to surgical excision. A. Recurrence-free survival in patients not receiving surgical excision, stratified by microabscess status. B: Recurrence-free survival in patients receiving surgical excision, stratified by microabscess status.

Since the microabscess-positive group had a higher rate of surgical excision compared to the microabscess-negative group (89.6% vs. 80.6%, P = 0.004; Table 2), patients were further stratified into four subgroups based on microabscess status and surgical excision status to assess whether surgical excision influenced the prognostic effect of microabscesses (Table 4). Survival analysis showed that among patients who did not undergo surgical excision, the microabscess-positive subgroup (e) had a lower one-year recurrence rate than the microabscess-negative subgroup (f) (21.5% vs. 43.3%, Fig 3A), but the difference was not statistically significant (P = 0.276). Similarly, among patients who underwent surgical excision, the microabscess-positive subgroup (g) also exhibited a lower one-year recurrence rate than the microabscess-negative subgroup (h) (20.0% vs. 25.7%, Fig 3B), although this difference also did not reach statistical significance (P = 0.081).

**Table 4:** Comparison of one-year recurrence rates by microabscess status and surgical excision.

group	surgical excision	microabscess	N	1-year recurrence rate(%)
e	no	positive	28	21.5
f	no	negative	49	43.3
g	yes	positive	240	20.0
h	yes	negative	203	25.7

### Therapeutic approach to microabscess

To further investigate optimal treatment strategies for microabscesses, this study compared the recurrence rates of various therapies between the microabscess-positive and microabscess-negative groups (Table 5). Several therapies, including penicillin, macrolide, and nitroimidazole therapies, were excluded due to insufficient sample size. It was found that antitubercular therapy significantly reduced the recurrence rate in both groups, whereas corticosteroid therapy was associated with an increased risk of recurrence. A notable difference was observed regarding abscess drainage: in the microabscess-positive group, patients who underwent drainage had a significantly lower one-year recurrence rate than those who did not (17.4% vs. 27.0%, P = 0.036; Table 5). However, in the microabscess-negative group, abscess drainage did not significantly affect recurrence (P = 0.652, Table 4). Furthermore, other treatments—including quinolone therapy, cephalosporin therapy, bromocriptine therapy, and surgical excision—did not improve prognosis in either patient group (Table 5).

**Table 5:** Comparison of recurrence rates by treatment strategy and microabscess status.

Factors	microabscess-positive group (N = 268)			microabscess-negative group (N = 252)		
	N(%)	1-year recurrence rate(%)	P value	N(%)	1-year recurrence rate(%)	P value
**Quinolone therapy**						
no	31(11.6)	22.8	0.690	51(20.2)	28.3	0.540
yes	237(88.4)	19.7		201(79.8)	28.7	
**Cephalosporin therapy**						
no	194(72.4)	20.9	0.211	186(73.8)	25.7	0.071
yes	74(27.6)	17.9		66(26.2)	37.4	
**Antitubercular therapy**						
no	172(64.2)	24.2	0.005	168(66.7)	34.4	0.010
yes	96(35.8)	13.4		84(33.3)	18.9	
**Corticosteroid therapy**						
no	78(29.1)	4.3	0.002	91(36.1)	17.5	0.004
yes	190(70.9)	25.4		161(63.9)	34.6	
**Bromocriptine therapy**						
no	147(54.9)	21.4	0.450	124(49.2)	33.3	0.812
yes	121(45.1)	18.7		128(50.8)	25.0	
**Abscess drainage**						
no	77(28.7)	27.0	0.036	75(29.8)	30.5	0.652
yes	191(71.3)	17.4		177(70.2)	27.8	
**Surgical excision**						
no	28(10.4)	21.5	0.719	49(19.4)	43.3	0.122
yes	240(89.6)	20.0		203(80.6)	25.7	

## Discussion

Our multivariate Cox regression across 520 well-characterized GLM patients identified five independent recurrence predictors: affected breast laterality, histologic microabscess, baseline white blood cell count, anti-mycobacterial medication, and corticosteroid use. Bilateral breast involvement carried the highest relapse hazard (HR = 7.864). Multiple prior single-center Chinese cohorts have reached the same conclusion: bilateral disease reflects widespread mammary inflammatory activity and multifocal hidden granulomas that local therapy cannot fully eradicate, making bilateral presentation a consistent high-risk marker [14]. Peripheral leukocytosis (≥10 × 10⁹/L) signals sustained systemic inflammation that drives repeated granuloma proliferation; clinicians should prioritize longer anti-inflammatory courses for patients with elevated baseline white blood cell counts [15].

Our most noteworthy finding runs counter to routine clinical intuition: microabscess visible on pathology independently lowers relapse risk (HR = 0.605, P = 0.010). Traditional clinical logic assumes any abscess correlates with worse inflammation and poorer long-term outcomes, yet our survival data contradict that assumption. Microabscess forms localized pockets that allow inflammatory debris and necrotic tissue to drain out of breast lobules, limiting diffuse granuloma spread. Patients without microabscess almost exclusively present as solid granulomatous masses with no natural drainage pathway for necrotic material; residual inflammatory tissue lingers and drives repeated flares [16]. In vitro and histologic work from Chen’s group at Wuhan University offers a mechanistic explanation: the primary GLM pathogen *Corynebacterium parakroppenstedtii* is microaerophilic, so the low-oxygen environment inside microabscess cavities suppresses sustained bacterial replication and reduces persistent inflammatory stimulus [17]. Notably, these mechanistic interpretations remain hypotheses, since we did not perform direct laboratory or microbiological experiments within this retrospective cohort. Further experimental work is required to formally validate these proposed biological pathways.

Anti-mycobacterial therapy strongly suppressed recurrence regardless of microabscess status (HR = 0.336, P < 0.001), consistent with the 2021 international multidisciplinary GLM consensus recommendations [12]. *Corynebacterium* strains linked to GLM share metabolic traits with mycobacteria, rendering standard penicillins and cephalosporins ineffective against dormant intralesional bacteria. Combination isoniazid, rifampicin and ethambutol targets latent microgranulomas and is appropriate for all patients with moderate or severe, recurrent GLM [18].

Conversely, corticosteroid use independently raised relapse risk (HR = 2.568, P = 0.001) in both microabscess subgroups. Steroids rapidly reduce swelling and pain by suppressing local macrophage function, but this blunts bacterial clearance. After tapering steroids, inflammation rebounds sharply, and prolonged steroid exposure increases fistula risk, as documented in multiple Chinese retrospective series [19,20]. Steroids should never be prescribed as monotherapy for GLM; any short steroid course must be paired with concurrent anti-infective or surgical intervention.

We noted two treatment imbalances at baseline: providers prescribed quinolones and performed full surgical resection more often for patients whose biopsy showed microabscess (P = 0.007 and P = 0.004). This likely reflects clinical intuition that microabscess signals active bacterial infection requiring aggressive combined therapy. We designed stratified survival analyses to isolate microabscess’s true prognostic effect independent of these treatment differences. The protective signal from microabscess only reached statistical significance in patients receiving quinolones (19.7% vs 28.7%, P = 0.030). We suspect quinolones’ high lipophilicity lets the drugs cross microabscess walls and clear intra-cavity Corynebacterium, amplifying the natural drainage benefit these tiny pus spaces provide. Solid granuloma masses without microabscess lack this permeable barrier, limiting quinolone tissue penetration and blunting their anti-relapse benefit [3]. One important limitation of this retrospective real‑world dataset is non‑randomized treatment allocation. Clinical teams selected therapeutic regimens partly guided by clinical severity, imaging and biopsy findings. Even though multivariate Cox regression and stratified subgroup analyses were used to adjust for measurable confounders, unmeasured indication bias and residual confounding cannot be fully excluded.

When we stratified by surgical resection status, microabscess still correlated with numerically lower recurrence in both resected and unresected patient groups, though neither comparison hit formal statistical thresholds (P = 0.276 and P = 0.081). Surgical resection cuts overall relapse rates for all GLM patients, but it does not erase the prognostic difference driven by histologic microabscess. Microabscess therefore acts as an independent pathologic biomarker whose predictive power holds separate from surgical intervention [21].

Drainage procedures (fine-needle aspiration, vacuum biopsy drainage, open debridement) reduced 1-year recurrence by nearly 10 percentage points for microabscess-positive patients (17.4% vs 27.0%, P = 0.036). For patients with purely solid granulomas and no microabscess on biopsy, drainage yielded no measurable relapse reduction (P = 0.652). Microabscess contains drainable pus and concentrated bacterial loads; removing this material interrupts granuloma expansion. Solid GLM lesions lack fluid collections, so drainage adds no therapeutic value and only creates unnecessary breast scarring. For patients without microabscess, clinicians should delay invasive drainage and use medication to shrink lesions before definitive resection [10,22].

Penicillins, macrolides and nitroimidazoles showed no consistent anti-relapse effect in either subgroup. Cephalosporins only showed a weak non-significant trend toward better outcomes for patients without microabscess (P = 0.071), so they are not our first-line recommendation. Quinolones deliver meaningful relapse protection specifically in patients with biopsy-proven microabscess, so levofloxacin or ciprofloxacin should be prioritized for this subgroup. For solid lesions without microabscess, cephalosporins may be used second-line, but long-term quinolone monotherapy is not advised [14].

Isoniazid, rifampicin and ethambutol combination lowered recurrence across both microabscess subgroups, making it the only treatment with consistent benefit regardless of histologic findings. We recommend a standard 9–12 month anti-mycobacterial course for all high-risk GLM presentations: bilateral disease, leukocytosis, repeated flares, or biopsy showing microabscess. Providers must monitor liver and retinal safety markers throughout long-term anti-mycobacterial treatment [18,23].

Steroid courses should be limited to short-term symptomatic control for severe diffuse erythema or secondary erythema nodosum, and never used as maintenance therapy. If steroids are unavoidable, concurrent quinolone or anti-mycobacterial coverage is mandatory, with gradual tapering to minimize inflammatory rebound [19,20].

## Study strengths and limitations

### Key strengths

This single-center cohort of 520 pathologically confirmed GLM patients represents one of the largest real-world Chinese datasets examining microabscess as a prognostic marker. Our standardized institutional registry reduced selection bias and guaranteed complete follow-up for all enrolled participants [13]. Unlike prior GLM studies, we built stratified survival models to test treatment-microabscess interaction effects, filling a gap in existing clinical guidance [7]. Our statistical workflow combined univariate screening, multivariate adjustment and multiple subgroup sensitivity analyses to control confounders, strengthening result reliability [6]. Every drug and surgical approach routinely used in domestic breast clinics was captured in our dataset, making our treatment recommendations directly applicable to Chinese clinical practice [1].

### Limitations

Because this work is a single‑center retrospective investigation, external validation via multi‑center prospective cohorts is essential to confirm generalizability of our microabscess‑related prognostic findings across different patient populations [6]. We did not collect records for traditional Chinese herbal medicines or immunosuppressants, so we cannot comment on how these alternative therapies perform across microabscess subgroups [7]. Our primary endpoint only tracked 1-year recurrence; extended follow-up out to 5 years would capture long-term relapse, breast deformity and contralateral new lesions [24]. We did not perform targeted microbial culture on microabscess tissue, which prevents us from drawing direct quantitative links between microabscess formation and intralesional Corynebacterium loads [3].

## Clinical takeaways and future research directions

Pathologic identification of microabscess offers a simple, readily available low-relapse biomarker for routine GLM workup. When biopsy confirms microabscess, we recommend combining full-course quinolones, complete abscess drainage and standard anti-mycobacterial therapy to minimize short-term relapse. Patients with solid granulomas and no microabscess should avoid routine drainage procedures, minimize steroid exposure, and delay surgery until medications shrink lesions to reduce cosmetic damage [23].

Several research lines warrant follow-up work. Multi-center prospective cohorts can build and externally validate a recurrence nomogram incorporating microabscess status [13]. Direct microbial culture of microabscess specimens can clarify how pus cavity formation correlates with pathogenic Corynebacterium counts [17]. Controlled trials comparing integrated Chinese and Western therapy can test herbal treatment efficacy split by microabscess subgroup [7]. Longitudinal 5-year follow-up will clarify how microabscess shapes long-term relapse risk and permanent breast structural damage [24].

## Conclusions

1. Histologically detected microabscess independently reduces 1-year recurrence risk for GLM patients. Bilateral breast involvement, elevated peripheral white blood cell counts and corticosteroid use all independently raise relapse risk, while standardized anti-mycobacterial medication consistently lowers recurrence across all patients [12,15].
2. Patients with microabscess receive more quinolone courses and more complete surgical resections at baseline. The protective effect of microabscess only reaches statistical significance among patients treated with quinolones; surgical resection reduces overall relapse rates but cannot eliminate the prognostic gap between microabscess subgroups [9,21].
3. Treatment efficacy differs sharply based on microabscess status. Abscess drainage only benefits patients with microscopic pus collections. Anti-mycobacterial triple therapy works for all GLM patients. Steroid monotherapy substantially increases relapse risk and should be restricted. Quinolones are the preferred first-line antibiotic for patients with biopsy-proven microabscess [10,18,19].
4. Clinicians can stratify GLM management using histologic microabscess results. For microabscess-positive patients, prioritize quinolone, drainage and anti-mycobacterial combination therapy. For microabscess-negative solid masses, skip unnecessary drainage, avoid steroid monotherapy, and time surgical resection optimally to preserve breast appearance [11,23].
