# Supplementary material for: Study on the influence of microabscess on the recurrence of granulomatous lobular mastitis and stratified treatment strategies
Source: PLoS One. 2026 Sep 23;21(9):e0358780. doi: 10.1371/journal.pone.0358780 (PMC13600525; doi:10.1371/journal.pone.0358780)
Supplement: Supplementary file 2 [file pone.0358780.s003.docx]

**Response to Reviewers**

**Manuscript ID: PONE‑D‑26‑32221**

Manuscript Title: Study on the Influence of Microabscess on the Recurrence of Granulomatous Lobular Mastitis and Stratified Treatment Strategies

Note: Page‑line numbers refer to the Revised Manuscript with Track Changes. All modifications are marked in the tracked‑change manuscript. We also submitted a clean unmarked manuscript, and all underlying anonymized study data are deposited publicly in Mendeley Data: <https://data.mendeley.com/datasets/5zpw78zrpk/1>.

**Response to Academic Editor’s Comments**

**Comment:** The authors state that "Existing theories link GLM onset to autoimmune dysfunction, elevated prolactin, hyperlipidemia, ductal injury, and infection with Corynebacterium species, often acting together". The authors should further clarify that Corynebacterium species are common constituents of the normal cutaneous microbiota, making the detection of these organisms in clinical specimens challenging to interpret and raising uncertainty regarding their clinical significance. This distinction is particularly important because isolation of Corynebacterium from breast tissue or secretions does not necessarily establish a causal role in GLM. Moreover, conventional culture‑based methods are relatively time‑consuming and may have limited sensitivity, potentially delaying identification of clinically relevant organisms. The authors should introduce these diagnostic limitations and consider whether more rapid and molecular approaches, e.g., metabolism‑driven sensor array, may help improve the detection and clinical interpretation of Corynebacterium in GLM. More reference should be cited, with these two (PMID: 34406882; PMID: 40737437) as examples (citing is optional).

**Response:** We sincerely appreciate this important comment. We have supplemented relevant text in the Introduction section to address this point.

Revised text added: “It should be noted that Corynebacterium species are common commensal components of human cutaneous microbiota. Therefore, isolating Corynebacterium from breast tissue or secretion samples cannot automatically confirm a causal pathogenic role for GLM. Conventional bacterial culture approaches are time‑consuming and have limited diagnostic sensitivity. Rapid molecular detection strategies, such as metabolism‑driven sensor arrays, may help improve pathogen identification and clinical interpretation of Corynebacterium‑associated mastitis.”

We also incorporated the suggested references (PMID: 34406882; PMID: 40737437) into our reference list.

**Comment:** Journal Requirements‑1. Please ensure that your manuscript meets PLOS ONE's style requirements, including those for file naming. The PLOS ONE style templates can be found at <https://journals.plos.org/plosone/s/file?id=wjVg/PLOSOne_formatting_sample_main_body.pdf> and <https://journals.plos.org/plosone/s/file?id=ba62/PLOSOne_formatting_sample_title_authors_affiliations.pdf>.

**Response:** We have reformatted the whole manuscript following PLOS ONE official formatting specifications: sentence‑case headings, proper figure‑table citation order, figure file naming (Fig1.tif, Fig2.tif etc.), table formatting, double‑spaced main text. Two manuscript versions are uploaded: Revised Manuscript with Track Changes and clean Manuscript. All figure files are submitted separately following journal graphic guidelines.

**Comment:** Journal Requirements‑2. In the online submission form you indicate that your data is not available for proprietary reasons and have provided a contact point for accessing this data. Please note that your current contact point is a co‑author on this manuscript. According to our Data Policy, the contact point must not be an author on the manuscript and must be an institutional contact, ideally not an individual. Please revise your data statement to a non‑author institutional point of contact, such as a data access or ethics committee, and send this to us via return email. Please also include contact information for the third‑party organization, and please include the full citation of where the data can be found.

**Response:** We thank you for pointing this out. All fully anonymized clinical dataset of this retrospective study has been deposited publicly in Mendeley Data (DOI:10.17632/5zpw78zrpk.1; <https://data.mendeley.com/datasets/5zpw78zrpk/1>). We updated the Data Availability Statement in manuscript and submission system.

Updated statement: “All anonymized clinical data supporting our findings are publicly available in Mendeley Data: Chen J (2026). Clinical and recurrence data of Granulomatous lobular mastitis (Sichuan, 2017‑2024), <https://data.mendeley.com/datasets/5zpw78zrpk/1>.”

We have also updated the corresponding field in Editorial Manager submission form.

**Comment:** Journal Requirements‑3. If the reviewer comments include a recommendation to cite specific previously published works, please review and evaluate these publications to determine whether they are relevant and should be cited. There is no requirement to cite these works unless the editor has indicated otherwise.

**Response:** We have carefully evaluated all suggested references. The two PMID references mentioned by academic editor are added to our reference list as described above.

**Comment:** Journal Requirements‑4. Please review your reference list to ensure that it is complete and correct. If you have cited papers that have been retracted, please include the rationale for doing so in the manuscript text, or remove these references and replace them with relevant current references. Any changes to the reference list should be mentioned in the rebuttal letter that accompanies your revised manuscript. If you need to cite a retracted article, indicate the article’s retracted status in the References list and also include a citation and full reference for the retraction notice.

**Response:** We have completed a full inspection of our reference list. No retracted articles are cited in this manuscript. All references are original relevant publications.

**Response to Reviewer #1**

**Comment‑1:** Authors should change the table formatting. Segregate the divisions in both columns and rows more clearly. The way presented the data is more unorganized. I would strongly suggest to at least bold the subheadings within the tables. For e.g. “Affected side” in the factor should be bold and left, right, bilateral comes under that.

**Response:** We thank the reviewer for this valuable suggestion. All major factor sub‑headings in Table1, Table2, Table4, Table5 are now formatted in bold font. Sub‑category items (left/right/bilateral etc.) are presented as indented plain text below bold main factor headers. Table borders and row‑column segmentation are optimized for readability according to PLOS ONE table guidelines. Changes are visible in tracked‑change manuscript.

**Comment‑2:** The authors should emphasize in the discussion that this is a single‑center retrospective study and that validation in multicenter or prospective cohorts would strengthen the generalizability of the findings.

**Response:** We have reinforced this statement in the Study Strengths and Limitations subsection of Discussion.

Added text: “Because this work is a single‑center retrospective investigation, external validation via multi‑center prospective cohorts is essential to confirm generalizability of our microabscess‑related prognostic findings across different patient populations.”

**Comment‑3:** I would also recommend discussion part as a paragraphed story rather as individual points.

**Response:** We have restructured the Discussion section, removing discrete subsection headings and reorganizing the whole content into continuous flowing paragraphs, while preserving all key logical layers: prognostic predictors, microabscess protective mechanism, baseline confounding, stratified treatment recommendations, strengths‑limitations, clinical take‑aways. The revised Discussion reads as a continuous narrative text.

**Comment‑4:** The discussion proposes possible biological mechanisms explaining why microabscesses may be associated with reduced recurrence. Since these mechanisms were not directly investigated in the present study, the authors should clearly indicate that these remain hypotheses requiring future experimental validation.

**Response:** We added explicit qualifying sentences in the mechanism paragraph of Discussion.

Added text: “Notably, these mechanistic interpretations remain hypotheses, since we did not perform direct laboratory or microbiological experiments within this retrospective cohort. Further experimental work is required to formally validate these proposed biological pathways.”

**Comment‑5:** Because treatment allocation was not randomized, clinicians may have selected different therapies based on disease severity. A brief discussion of potential indication bias and residual confounding would be good.

**Response:** We added a paragraph addressing indication bias and residual confounding within Discussion.

Added text: “One important limitation of this retrospective real‑world dataset is non‑randomized treatment allocation. Clinical teams selected therapeutic regimens partly guided by clinical severity, imaging and biopsy findings. Even though multivariate Cox regression and stratified subgroup analyses were used to adjust for measurable confounders, unmeasured indication bias and residual confounding cannot be fully excluded.”

**Overall assessment:** This manuscript presents a valuable retrospective clinical study investigating the prognostic significance of histologic microabscess in granulomatous lobular mastitis. The study is based on a substantial patient cohort, employs appropriate statistical analyses, and provides clinically relevant findings that may assist in individualized treatment planning. The conclusions are supported by the presented data. I recommend acceptance after minor editorial revisions.

**Response:** Thank you very much for your positive evaluation and constructive suggestions. All above‑mentioned revisions have been implemented in the revised manuscript.

**Response to Reviewer #2**

**Comment:** I have carefully reviewed the manuscript and find that it is well organized, scientifically sound, and clearly written. The objectives are well defined, the methodology is appropriate, and the results are presented and discussed logically. The conclusions are supported by the data presented. The manuscript makes a valuable contribution to the field and is suitable for publication. I recommend acceptance in its current form, with no further revisions required.

**Response:** We sincerely appreciate your positive assessment of our study. We have implemented all revisions requested by academic editor and Reviewer #1, without altering the core data, results and conclusions of this work.

**Additional submission checklist (as requested by journal)**

1. Separate files uploaded: ‑ Response to Reviewers (this document) ‑ Revised Manuscript with Track Changes ‑ Clean Manuscript (no tracked changes) ‑ Individual figure files (Fig1.tif, Fig2.tif, Fig3.tif)
2. Dataset public repository: Mendeley Data DOI:10.17632/5zpw78zrpk.1
3. Institutional non‑author contact for data access updated in manuscript and submission form.
4. ORCID information for corresponding author is verified in Editorial Manager.

We hope the revised manuscript now meets all publication criteria for PLOS ONE. We are happy to provide further modifications if additional comments arise.

Sincerely,

Corresponding Author: Haiyan Zhang

On behalf of all co‑authors
