# Supplementary material for: Study on the influence of microabscess on the recurrence of granulomatous lobular mastitis and stratified treatment strategies
Source: PLoS One. 2026 Sep 23;21(9):e0358780. doi: 10.1371/journal.pone.0358780 (PMC13600525; doi:10.1371/journal.pone.0358780)
Supplement: Supplementary file 3 [file pone.0358780.s001.docx]

**Major strengths**

The study includes 520 pathologically confirmed GLM patients collected over several years with complete follow-up, providing adequate statistical power for prognostic analyses. The identification of histologic microabscess as a potential predictor of recurrence addresses an important clinical question for disease management and long-term follow-up. The use of multivariable Cox proportional hazards regression together with Kaplan–Meier survival analysis is appropriate for evaluating recurrence risk and treatment outcomes. The manuscript not only identifies prognostic factors but also evaluates multiple therapeutic approaches, including quinolones, anti-mycobacterial therapy, corticosteroids, abscess drainage, and surgical excision, providing practical clinical information. The manuscript is clearly structured, and the figures and tables effectively summarize the results. The discussion appropriately places the findings within the context of current literature.

**Minor comments**

- Authors should change the table formatting. Segregate the divisions in both columns and rows more clearly. The way presented the data is more unorganized. I would strongly suggest to at least bold the subheadings within the tables. For e.g. “Affected side” in the factor should be bold and left, right, bilateral comes under that.
- The authors should emphasize in the discussion that this is a single-center retrospective study and that validation in multicenter or prospective cohorts would strengthen the generalizability of the findings.
- I would also recommend discussion part as a paragraphed story rather as individual points.
- The discussion proposes possible biological mechanisms explaining why microabscesses may be associated with reduced recurrence. Since these mechanisms were not directly investigated in the present study, the authors should clearly indicate that these remain hypotheses requiring future experimental validation.
- Because treatment allocation was not randomized, clinicians may have selected different therapies based on disease severity. A brief discussion of potential indication bias and residual confounding would be good.

**Overall assessment**

This manuscript presents a valuable retrospective clinical study investigating the prognostic significance of histologic microabscess in granulomatous lobular mastitis. The study is based on a substantial patient cohort, employs appropriate statistical analyses, and provides clinically relevant findings that may assist in individualized treatment planning. The conclusions are supported by the presented data, and the manuscript represents a useful contribution to the literature on GLM management.

I recommend **acceptance after minor editorial revisions**.

**Recommendation: Accept with Minor Revisions.**
