# Supplementary material for: Study on the influence of microabscess on the recurrence of granulomatous lobular mastitis and stratified treatment strategies
Source: PLoS One. 2026 Sep 23;21(9):e0358780. doi: 10.1371/journal.pone.0358780 (PMC13600525; doi:10.1371/journal.pone.0358780)
Supplement: Peer review history

# Peer review: Decision Letter 0

**DOI:** 10.1371/journal.pone.0358780.r001

**Authors:** Benjamin Liu

9 Aug 2026

Dear Dr. chen,

Thank you for submitting your manuscript to PLOS ONE. After careful consideration, we feel that it has merit but does not fully meet PLOS ONE’s publication criteria as it currently stands. Therefore, we invite you to submit a revised version of the manuscript that addresses the points raised during the review process.

================================

**ACADEMIC EDITOR'S COMMENTS:**

================================

Please submit your revised manuscript by Sep 08 2026 11:59PM. If you will need more time than this to complete your revisions, please reply to this message or contact the journal office at plosone@plos.org. When you're ready to submit your revision, log on to https://www.editorialmanager.com/pone/ and select the 'Submissions Needing Revision' folder to locate your manuscript file.

- A letter that responds to each point raised by the academic editor and reviewer(s). You should upload this letter as a separate file labeled 'Response to Reviewers'.
- A marked-up copy of your manuscript that highlights changes made to the original version. You should upload this as a separate file labeled 'Revised Manuscript with Track Changes'.
- An unmarked version of your revised paper without tracked changes. You should upload this as a separate file labeled 'Manuscript'.

If you would like to make changes to your financial disclosure, please include your updated statement in your cover letter. Guidelines for resubmitting your figure files are available below the reviewer comments at the end of this letter.

If applicable, we recommend that you deposit your laboratory protocols in protocols.io to enhance the reproducibility of your results. Protocols.io assigns your protocol its own identifier (DOI) so that it can be cited independently in the future. For instructions see: https://journals.plos.org/plosone/s/submission-guidelines#loc-laboratory-protocols. Additionally, PLOS ONE offers an option for publishing peer-reviewed Lab Protocol articles, which describe protocols hosted on protocols.io. Read more information on sharing protocols at https://plos.org/protocols?utm_medium=editorial-email&utm_source=authorletters&utm_campaign=protocols.

As the corresponding author, your ORCID iD is verified in the submission system and will appear in the published article. PLOS supports the use of ORCID, and we encourage all coauthors to register for an ORCID iD and use it as well. Please encourage your coauthors to verify their ORCID iD within the submission system before final acceptance, as unverified ORCID iDs will not appear in the published article. *Only*  the individual author can complete the verification step; PLOS staff *cannot*  verify ORCID iDs on behalf of authors.

We look forward to receiving your revised manuscript.

Kind regards,

Benjamin M. Liu, PhD, D(ABMM), MB(ASCP)

Academic Editor

PLOS One

**Journal Requirements:**

When submitting your revision, we need you to address these additional requirements.

1. Please ensure that your manuscript meets PLOS ONE's style requirements, including those for file naming. The PLOS ONE style templates can be found at

https://journals.plos.org/plosone/s/file?id=wjVg/PLOSOne_formatting_sample_main_body.pdf and

https://journals.plos.org/plosone/s/file?id=ba62/PLOSOne_formatting_sample_title_authors_affiliations.pdf

2. In the online submission form you indicate that your data is not available for proprietary reasons and have provided a contact point for accessing this data. Please note that your current contact point is a co-author on this manuscript. According to our Data Policy, the contact point must not be an author on the manuscript and must be an institutional contact, ideally not an individual. Please revise your data statement to a non-author institutional point of contact, such as a data access or ethics committee, and send this to us via return email. Please also include contact information for the third party organization, and please include the full citation of where the data can be found.

3. If the reviewer comments include a recommendation to cite specific previously published works, please review and evaluate these publications to determine whether they are relevant and should be cited. There is no requirement to cite these works unless the editor has indicated otherwise.

4. Please review your reference list to ensure that it is complete and correct. If you have cited papers that have been retracted, please include the rationale for doing so in the manuscript text, or remove these references and replace them with relevant current references. Any changes to the reference list should be mentioned in the rebuttal letter that accompanies your revised manuscript. If you need to cite a retracted article, indicate the article’s retracted status in the References list and also include a citation and full reference for the retraction notice.

[Note: HTML markup is below. Please do not edit.]

Reviewers' comments:

Reviewer's Responses to Questions

**Comments to the Author**

1. Is the manuscript technically sound, and do the data support the conclusions?

Reviewer #1: Yes

Reviewer #2: Yes

**********

2. Has the statistical analysis been performed appropriately and rigorously?

Reviewer #1: Yes

Reviewer #2: Yes

**********

3. Have the authors made all data underlying the findings in their manuscript fully available?

The PLOS Data policy

Reviewer #1: Yes

Reviewer #2: Yes

**********

4. Is the manuscript presented in an intelligible fashion and written in standard English?

Reviewer #1: Yes

Reviewer #2: Yes

**********

Reviewer #1: The study includes 520 pathologically confirmed GLM patients collected over several years with complete follow-up, providing adequate statistical power for prognostic analyses. The identification of histologic microabscess as a potential predictor of recurrence addresses an important clinical question for disease management and long-term follow-up. The use of multivariable Cox proportional hazards regression together with Kaplan–Meier survival analysis is appropriate for evaluating recurrence risk and treatment outcomes. The manuscript not only identifies prognostic factors but also evaluates multiple therapeutic approaches, including quinolones, anti-mycobacterial therapy, corticosteroids, abscess drainage, and surgical excision, providing practical clinical information. The manuscript is clearly structured, and the figures and tables effectively summarize the results. The discussion appropriately places the findings within the context of current literature.

Minor comments

•Authors should change the table formatting. Segregate the divisions in both columns and rows more clearly. The way presented the data is more unorganized. I would strongly suggest to at least bold the subheadings within the tables. For e.g. “Affected side” in the factor should be bold and left, right, bilateral comes under that.

•The authors should emphasize in the discussion that this is a single-center retrospective study and that validation in multicenter or prospective cohorts would strengthen the generalizability of the findings.

•I would also recommend discussion part as a paragraphed story rather as individual points.

•The discussion proposes possible biological mechanisms explaining why microabscesses may be associated with reduced recurrence. Since these mechanisms were not directly investigated in the present study, the authors should clearly indicate that these remain hypotheses requiring future experimental validation.

•Because treatment allocation was not randomized, clinicians may have selected different therapies based on disease severity. A brief discussion of potential indication bias and residual confounding would be good.

Overall assessment

This manuscript presents a valuable retrospective clinical study investigating the prognostic significance of histologic microabscess in granulomatous lobular mastitis. The study is based on a substantial patient cohort, employs appropriate statistical analyses, and provides clinically relevant findings that may assist in individualized treatment planning. The conclusions are supported by the presented data, and the manuscript represents a useful contribution to the literature on GLM management.

I recommend acceptance after minor editorial revisions.

Recommendation: Accept with Minor Revisions.

Reviewer #2: I have carefully reviewed the manuscript and find that it is well organized, scientifically sound, and clearly written. The objectives are well defined, the methodology is appropriate, and the results are presented and discussed logically. The conclusions are supported by the data presented.

The manuscript makes a valuable contribution to the field and is suitable for publication. I recommend acceptance in its current form, with no further revisions required.

**********

what does this mean?). If published, this will include your full peer review and any attached files.

If you choose “no”, your identity will remain anonymous but your review may still be made public.

**Do you want your identity to be public for this peer review?**  For information about this choice, including consent withdrawal, please see our Privacy Policy

Reviewer #1: **Yes:**  Kishor Jakkala

Reviewer #2: No

**********

[NOTE: If reviewer comments were submitted as an attachment file, they will be attached to this email and accessible via the submission site. Please log into your account, locate the manuscript record, and check for the action link "View Attachments". If this link does not appear, there are no attachment files.]

To ensure your figures meet our technical requirements, please review our figure guidelines: https://journals.plos.org/plosone/s/figures

You may also use PLOS’s free figure tool, NAAS, to help you prepare publication quality figures: https://journals.plos.org/plosone/s/figures#loc-tools-for-figure-preparation.

NAAS will assess whether your figures meet our technical requirements by comparing each figure against our figure specifications.

Attachment Submitted filename: Review report_PlosOne.docx

# Author comment: Author response to Decision Letter 1

**DOI:** 10.1371/journal.pone.0358780.r002

28 Aug 2026

Dear PLOS ONE Editorial Office,

We have completed all revisions according to your technical requirements:

1. All outdated manuscript files have been removed from the submission file inventory, only the latest documents are retained.

2. Two versions of revised manuscript are uploaded: the clean manuscript without tracked changes (marked as "Manuscript"), and the version with tracked changes (file type set as "Revised Manuscript w/tracked changes").

3. All embedded figure images have been deleted from the manuscript word file. Figure legends are kept in text. Individual TIFF figure files are uploaded separately.

We have reviewed and approved the rebuilt submission PDF. Please proceed with further editorial processing.

Thank you very much.

Best regards,

Corresponding author

Attachment Submitted filename: Response to Reviewers.docx

# Editor report: Decision Letter 1

**DOI:** 10.1371/journal.pone.0358780.r003

**Authors:** Benjamin Liu

6 Sep 2026

Study on the Influence of Microabscess on the Recurrence of Granulomatous Lobular Mastitis and Stratified Treatment Strategies

PONE-D-26-32221R1

Dear Dr. Chen,

We’re pleased to inform you that your manuscript has been judged scientifically suitable for publication and will be formally accepted for publication once it meets all outstanding technical requirements.

Within one week, you’ll receive an e-mail detailing the required amendments. When these have been addressed, you’ll receive a formal acceptance letter and your manuscript will be scheduled for publication.

An invoice will be generated when your article is formally accepted. Please note, if your institution has a publishing partnership with PLOS and your article meets the relevant criteria, all or part of your publication costs will be covered. Please make sure your user information is up-to-date by logging into Editorial Manager at Editorial Manager® and clicking the ‘Update My Information' link at the top of the page. For questions related to billing, please contact billing support.

If your institution or institutions have a press office, please notify them about your upcoming paper to help maximize its impact. If they’ll be preparing press materials, please inform our press team as soon as possible -- no later than 48 hours after receiving the formal acceptance. Your manuscript will remain under strict press embargo until 2 pm Eastern Time on the date of publication. For more information, please contact onepress@plos.org.

Kind regards,

Benjamin M. Liu, PhD, D(ABMM), MB(ASCP)

Academic Editor

PLOS One

Additional Editor Comments (optional):

Reviewers' comments:

# Editor report: Acceptance letter

**DOI:** 10.1371/journal.pone.0358780.r004

**Authors:** Benjamin Liu

PONE-D-26-32221R1

PLOS One

Dear Dr. chen,

I'm pleased to inform you that your manuscript has been deemed suitable for publication in PLOS One. Congratulations! Your manuscript is now being handed over to our production team.

At this stage, our production department will prepare your paper for publication. This includes ensuring the following:

* All references, tables, and figures are properly cited

* All relevant supporting information is included in the manuscript submission,

* There are no issues that prevent the paper from being properly typeset

You will receive further instructions from the production team, including instructions on how to review your proof when it is ready. Please keep in mind that we are working through a large volume of accepted articles, so please give us a few days to review your paper and let you know the next and final steps.

Lastly, if your institution or institutions have a press office, please let them know about your upcoming paper now to help maximize its impact. If they'll be preparing press materials, please inform our press team within the next 48 hours. Your manuscript will remain under strict press embargo until 2 pm Eastern Time on the date of publication. For more information, please contact onepress@plos.org.

You will receive an invoice from PLOS for your publication fee after your manuscript has reached the completed accept phase. If you receive an email requesting payment before acceptance or for any other service, this may be a phishing scheme. Learn how to identify phishing emails and protect your accounts at https://explore.plos.org/phishing.

If we can help with anything else, please email us at customercare@plos.org.

Thank you for submitting your work to PLOS One and supporting open access.

Kind regards,

PLOS One Editorial Office Staff

on behalf of

Dr. Benjamin M. Liu

Academic Editor

PLOS One
